# New Peptides Isolated from Marine Cyanobacteria, an Overview over the Past Decade

**DOI:** 10.3390/md15050132

**Published:** 2017-05-05

**Authors:** Yue Mi, Jinrong Zhang, Shan He, Xiaojun Yan

**Affiliations:** 1 School of Marine Sciences, Laboratory of Marine Natural Products, Ningbo University, Ningbo 315211, China; yuemi19952017@163.com (Y.M.); heshan@nbu.edu.cn (S.H.); 2 Key Laboratory of Applied Marine Biotechnology of Ministry of Education, Ningbo University, Ningbo 315211, China; yanxiaojun@nbu.edu.cn

**Keywords:** marine cyanobacteria, peptide, secondary metabolites, bioactivity

## Abstract

Marine cyanobacteria are significant sources of structurally diverse marine natural products with broad biological activities. In the past 10 years, excellent progress has been made in the discovery of marine cyanobacteria-derived peptides with diverse chemical structures. Most of these peptides exhibit strong pharmacological activities, such as neurotoxicity and cytotoxicity. In the present review, we summarized peptides isolated from marine cyanobacteria since 2007.

## 1. Introduction

Cyanobacteria, as some of the oldest aquatic and photosynthetic oxygenic prokaryotes, are widely distributed in the world [1]. In recent years, cyanobacteria from different habitats, particularly marine cyanobacteria, are found to contain a great deal of bioactive secondary metabolites. As chemical defenses, secondary metabolites from marine cyanobacteria can improve adaptability of marine cyanobacteria to various marine environments, which are characterized by hyperhaline, high-pressure, barren and complexity. These secondary metabolites from marine cyanobacteria not only greatly affect the growth and reproduction of cyanobacteria, but also show many biological activities, such as anti-tumor, antibacterial, enzyme inhibition, parasitic resistance, anti-inflammatory and other biological activities [2]. Therefore, they have attracted extensive attention of scholars in different subject fields, such as medicinal chemistry, pharmacology and marine chemical ecology. It is considered that more drug lead compounds can be found from marine cyanobacteria through the cooperative in-depth and systematic studies. Marine cyanobacteria-derived bioactive components have gained great popularity in research on marine natural products [3]. For example, dolastatin 10 analogue soblidotin (or TZT-1027, auristatin PE) has shown a good prospect in human colonic carcinoma, which has been developed to the phase II clinical trials [4]. Brentuximab vedotin (trade name Adcetris), a marine peptide-derived drug, was approved by the U.S. Food and Drug Administration (FDA) in 2011 for cancer treatment [5]. In the past 10 years (from 2007 to 2016), the programs for drug discovery from marine cyanobacteria, such as Panama International Cooperative Biodiversity Group (ICGB) program, have discovered more than 400 new natural compounds from marine cyanobacteria. Among these compounds, peptides and peptide-containing compounds are the major secondary metabolites.

In 2006, secondary metabolites from marine cyanobacteria were reviewed [6]. Biological targets and the mechanisms of action of bioactive natural products from marine cyanobacteria were also reviewed in 2015 [7]. In the present review, we systematically reviewed the current progress on the discovery of peptides and peptide-containing compounds from marine cyanobacteria since 2007. By the end of 2016, a total of 126 new peptide-compounds have been isolated from marine cyanobacteria, mainly from the genera *Lyngbya*, *Oscillatoria* and *Symploca*. However, two new genera *Moorea* and *Okeania*, which were previously identified as the polyphyletic cyanobacterial genus *Lyngbya*, have been proposed using genome sequence analysis in the past few years [8,9]. Another new genus *Caldora*, which was previously identified as *Symploca*, has also been proposed [10]. New peptide compounds, which were actually isolated from these new genera of cyanobacteria, were also mentioned in this review. Most of these peptides with chemical diversity exhibit strong biological activities, such as neurotoxicity and cytotoxicity. In addition, cyclic depsipeptides, including 76 compounds, are the main cyclic peptides discovered from marine cyanobacteria.

## 2. Linear Peptides

Linear peptides are common compounds isolated from marine cyanobacteria, most of which exhibit prodigious biological activities, such as anti-tumor, antimicrobial, antimalarial, enzyme inhibition and other biological activities [11]. Table 1 and Table 2 summarize 39 linear peptides derived from marine cyanobacteria, covering literature from January 2007 to December 2016.

### 2.1. Linear Depsipeptides

**Figure 1:**
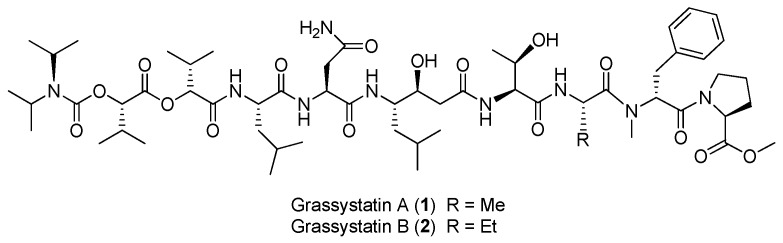

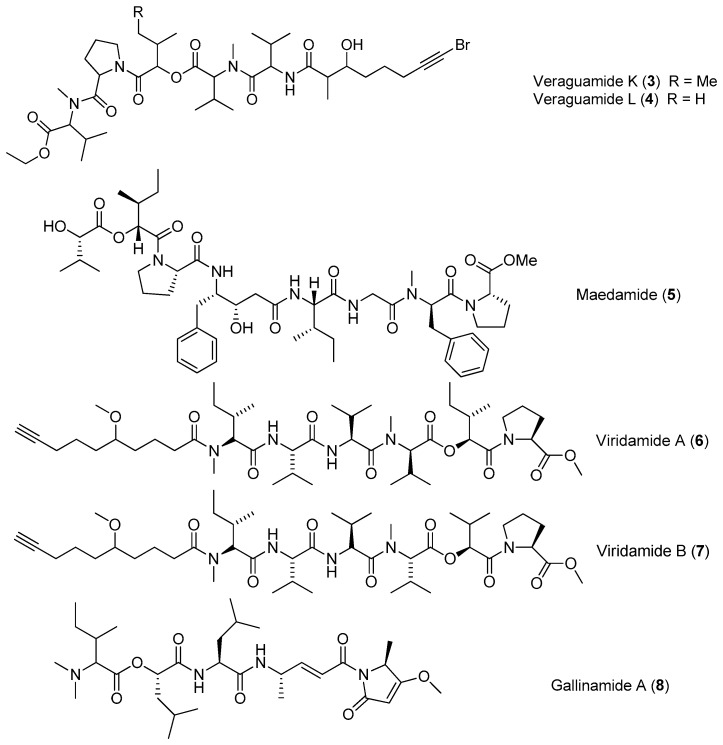
Chemical structures of compounds **1**–**8**.

Two linear depsipeptides, grassystatins A and B (**1**, **2**), have been purified from the marine cyanobacterium *Okeania lorea* (formerly *Lyngbya* cf. *confervoides*) collected from Key Largo [9], Florida (Figure 1). Compound **1** displays selectivity against cathepsins D and E with IC_50_ values of 26.5 nM and 886 pM, respectively. Compound **2** can also selectively inhibit cathepsins D and E with IC_50_ values of 7.27 nM and 354 pM, respectively. Selective inhibition of **1**–**2** against cathepsin E over cathepsin D (20- to 38-fold) suggests that these bioactive compounds **1** and **2** are useful tools to probe cathepsin E function [12]. Moreover, the total synthesis of **1** has been completed [13].

Two bromide-containing linear depsipeptides, veraguamides K–L (**3**–**4**), have been discovered from marine cyanobacterium cf. *Oscillatoria margaritifera* collected from Coiba Island National Park, Panama, which are supposed to exhibit the structural characteristics of marine natural products [14]. Maedamide (**5**) has been extracted from marine cyanobacterial assemblage of *Lyngbya* sp., which shows strong and selective inhibition against chymotrypsin (IC_50_ value of 45 μM), but not against elastase or trypsin. Moreover, compound **5** inhibits the growth of Hela cells and HL60 cells (IC_50_ values of 4.2 and 2.2 μM, respectively) and induces apoptosis in Hela cells [15]. The total synthesis of **5** has been achieved, leading to reassignment of the structure of **5** [16].

Two PKS-NRPS-derived metabolites, viridamides A, B (**6**, **7**), have been discovered from the marine cyanobacterium *Okeania comitata* (formerly *Oscillator nigroviridis*) collected from Panama [9]. Compound **6** shows anti-trypanosomal and antileishmanial activities with IC_50_ values of 1.1 and 1.5 μM, respectively [17]. An antimalarial peptide, termed gallinamide A (**8**), has been purified from Panamanian marine cyanobacteria, showing moderate antimalarial activity against chloroquine-resistant strain (W2) of *Plasmodium falciparum* (IC_50_ = 8.4 μM) [18]. Total synthesis of compound **8** has been completed [19].

### 2.2. Other Linear Peptides

**Figure 2:**
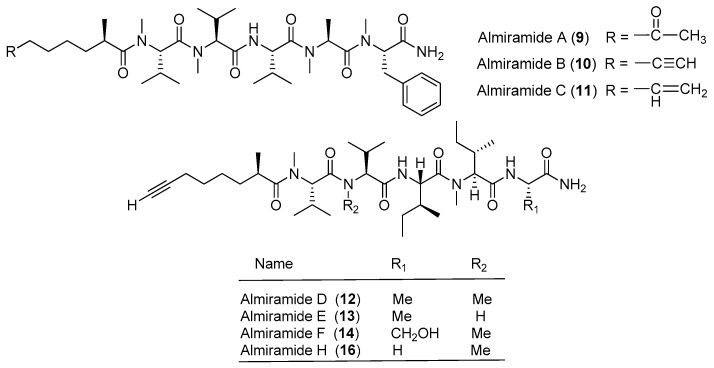

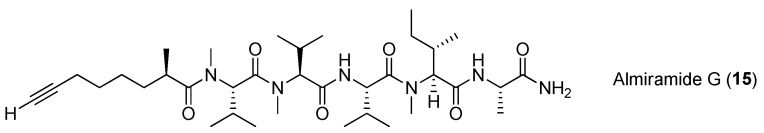
Chemical structures of compounds **9**–**16**.

Three highly *N*-methylated linear lipopeptides, almiramides A–C (**9**–**11**), have been identified from screening of the marine cyanobacterium *Lyngbya majuscula* collected from Panama for antiparasitic activities against *Leishmaniasis donovani* (Figure 2). Compounds **9**, **10** and **11** display strong antileishmanial activity with IC_50_ values of 13.5, 2.4 and 1.9 μM, respectively [20].

Five analogues of compound **9**, almiramides D–H (**12**–**16**), have been derived from the marine cyanobacterium *Oscillatoria nigroviridis* collected from the Providence Island, Colombian Caribbean Sea. Compounds **10** and **12** exhibit mild toxicity against five human tumor cell lines (A549, MCF-7, HeLa, PC3 and MDA-MB231) and high toxicity against the gingival fibroblast cell line [21].

**Figure 3:**
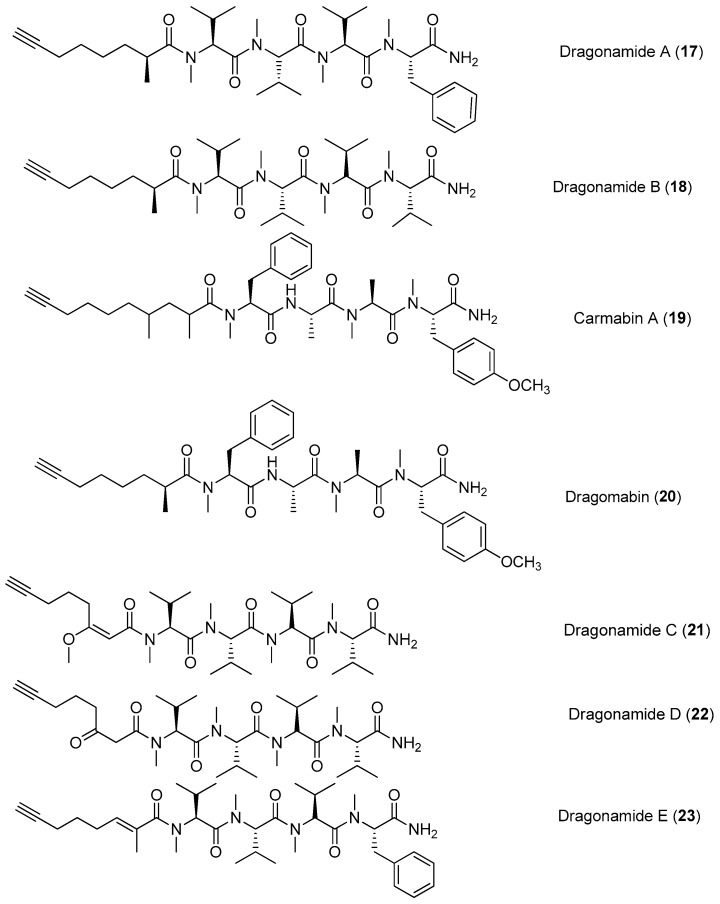
Chemical structures of compounds **17**–**23**.

Four lipopeptides, named dragonamides A and B (**17**, **18**), carmabin A (**19**) and dragomabin (**20**), have been identified from the antimalarial bioassay-guided isolation of the marine cyanobacterium *Moorea producens* (formerly *Lyngbya majuscula*) (Figure 3). Compounds **17**, **19** and **20** exhibit good antimalarial activity (IC_50_ = 7.7, 4.3 and 6.0 μM, respectively) [8,22]. Two analogs of **17**, named dragonamides C and D (**21**, **22**), have been isolated from the marine cyanobacterium *Moorea producens* (formerly *Lyngbya polychroa*) collected from Hollywood Beach, Fort Lauderdale, Florida. Compounds **21** and **22** display weak cytotoxicity in cancer cell viability assays [23,24]. A new antimalarial peptide, termed dragonamide E (**23**), has been purified from the marine cyanobacterium *Lyngbya majuscula* and shows antileishmanial activity with an IC_50_ value of 5.1 μM [25].

**Figure 4:**
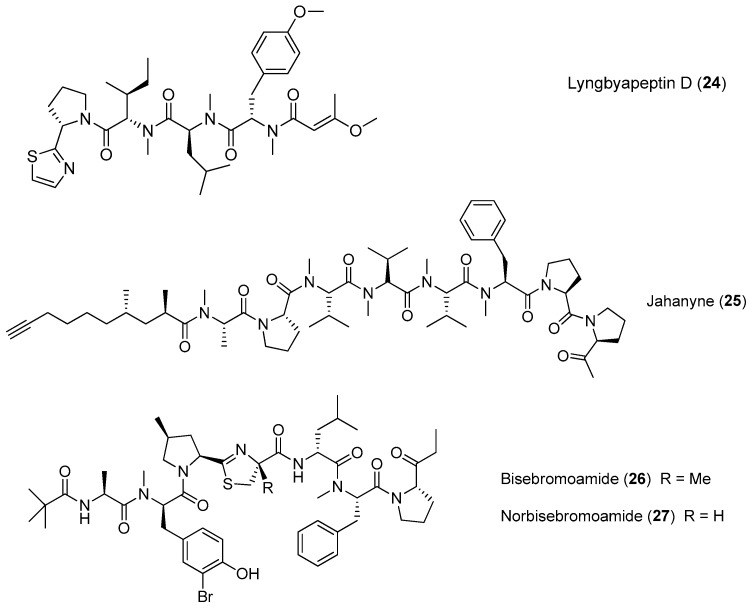
Chemical structures of compounds **24**–**27**.

A linear lipopeptide, lyngbyapeptin D (**24**), has been purified from the marine cyanobacterium *Moorea bouillonii* (formerly *Lyngbya bouillonii*) collected from Apra Harbor, Guam [8,26]. Jahanyne (**25**), isolated from *Lyngbya* sp. collected in Okinawa, shows significant inhibitory effects on the growth of human cancer cells in vitro, and it can induce apoptosis of HeLa cells [27]. Two novel cytotoxic peptides, named bisebromoamide (**26**) and norbisebromoamide (**27**), have been identified from the marine cyanobacterium *Lyngbya* sp. (Figure 4). The rare peptide **26** possesses the combination of unusual structural features, including an *N*-pivalamide moiety, high degree of *D*-amino acids, *N*-methylated amino acids and several other modified amino acid residues of nonribosomal origin. Compound **26** exhibits cytotoxicity against HeLa S3 cells (IC_50_ = 0.04 μg/mL). Compound **26** can also inhibit phosphorylation of ERK (extracellular signal regulated protein kinase) in NRK cells, showing potent and selective inhibitory effects on protein kinase [28,29]. A revised configurational assignment for the marine peptide **26** has been proposed and validated by total synthesis [30,31].

**Figure 5:**
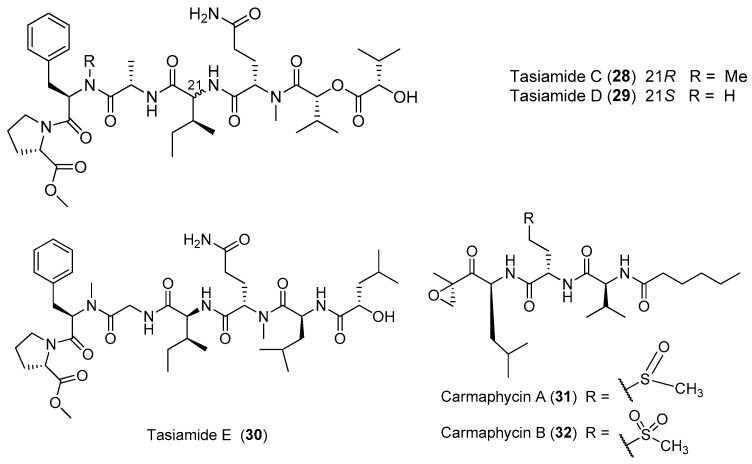
Chemical structures of compounds **28**–**32**.

Three new lipopeptides, tasiamides C–E (**28**–**30**), have been derived from the tropical marine cyanobacterium *Symploca* sp. collected near Kimbe Bay, Papua New Guinea (Figure 5). The structural features of **28**–**30** are similar to some previously isolated peptides from the same marine cyanobacterium *Symploca* sp., such as tasiamides, grassystatins and symplocin [32]. Two novel proteasome inhibitors, carmaphycins A and B (**31**, **32**), have been extracted from the marine cyanobacterium *Symploca* sp. collected from Curacao, and both of them possess a leucine-derived *α*, *β*-epoxyketone directly connected to either a sulfoxide or sulfone moiety. Compounds **31** and **32** strongly inhibit the *β*5 subunit of the *S. cerevisiae* 20S proteasome and show strong cytotoxicity against the lung and colon cancer cells. The total synthesis of **31** and **32** has been accomplished [33].

**Figure 6:**
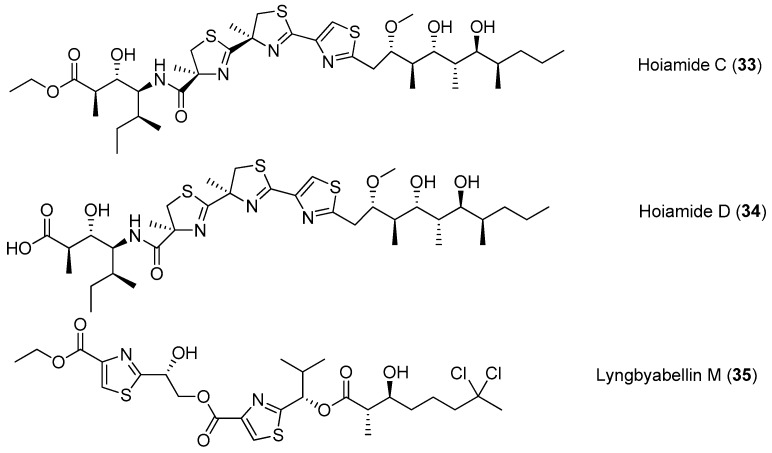
Chemical structures of compounds **33**–**35**.

A structurally intriguing neurotoxic lipopeptide, hoiamide C (**33**), has been extracted from marine cyanobacteria collected in Papua New Guinea, and it possesses unique structural features of *S*-adenosyl methionine modified isoleucine unit, a central triheterocyclic system consisting of two R-methylated thiazolines and one thiazole moiety [34]. The total synthesis of **33** has been accomplished [35]. Hoiamide D (**34**), a new analogue of compound **33**, has been purified from two cyanobacteria by bioassay-guided isolation (Figure 6). Compound **34** shows strong inhibitory activity against an attractive anticancer target p53/MDM2 interaction (EC_50_ = 4.5 μM) [36]. Another thiazole-containing lipopeptide, lyngbyabellin M (**35**), has been extracted from the cyanobacterium *Moorea bouillonii* from Palmyra Atoll, Central Pacific Ocean [37].

**Figure 7:**
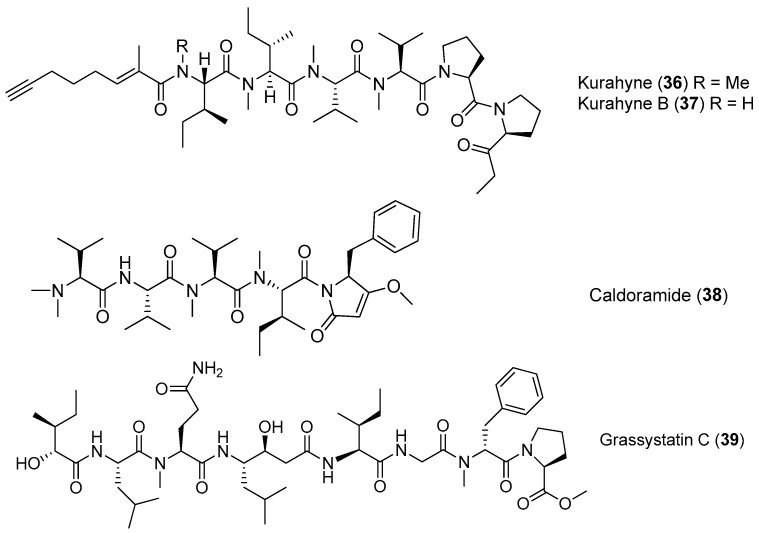
Chemical structures of compounds **36**–**39**.

A new acetylene-containing lipopeptide, named Kurahyne (**36**), has been isolated from the cyanobacterial mixture consisting of *Lyngbya* sp. mostly. Compound **36** shows the inhibition against the growth of human cancer cells and induces the apoptosis of HeLa cells [38]. A new analogue of **36**, kurahyne B (**37**), has been identified from the marine cyanobacterium *Okeania* sp. from Okinawa. Compound **37** inhibits the growth of HeLa cells and HL60 cells with IC_50_ values of 8.1 and 9.0 μM, respectively [39]. A cytotoxic pentapeptide caldoramide (**38**) has been extracted from the marine cyanobacterium *Caldora penicillata* from Big Pine Key, Florida (Figure 7). Compound **38** shows differential cytotoxicity against parental HCT116 colorectal cancer cells and isogenic cells lacking oncogenic KRAS or hypoxia-inducible factors 1*α* (HIF-1*α*) and 2*α* (HIF-2*α*) [40]. A linear peptide, grassystatin C (**39**), has been purified from the marine cyanobacterium *Okeania lorea* (formerly *Lyngbya* cf. *confervoides*) collected from Key Largo, Florida [9]. Compound **39**, which consists of two fewer residues compared with **1** and **2**, is less effective against both cathepsins D and E [12].

## 3. Cyclic Peptides

Cyclic peptides are a class of natural products with structural diversity and pharmacological perspective. In the past 10 years, the discovery of bioactive marine natural products from marine cyanobacteria has become a new research hotpoint in the field of marine natural products since a large number of bioactive cyclic peptides have been derived from marine cyanobacteria. The structural characteristics of cyclic peptides from marine cyanobacteria mainly manifest in tremendous diversity in new carbon skeletons, oxidation of the carbon skeletons of amino acids, complexes of holagen-containing molecules and complex spatial configuration [6]. Cyclic peptides from marine cyanobacteria can be further divided into cyclic depsipeptides, cyclic liopeptides and other cyclic peptides. A total of 87 cyclic peptides isolated from marine cyanobacteria, covering literature from January 2007 to December 2016, are summarized in Table 3, Table 4 and Table 5.

### 3.1. Cyclic Depsipeptides

**Figure 8:**
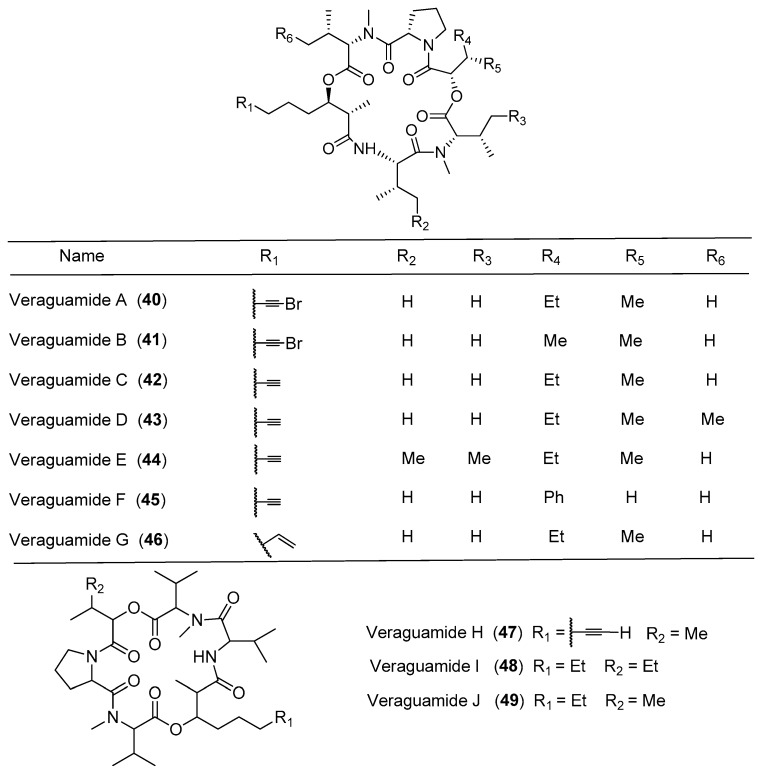
Chemical structures of compounds **40**–**49**.

Seven new cyclic hexadepsipeptides, termed veraguamides A–G (**40**–**46**), have been identified through cytotoxicity-directed isolation of a marine cyanobacterium *Symploca* cf. *hydnoides* sample from Cetti Bay, Guam (Figure 8). Compounds **40**–**46** show moderate to weak cytotoxicity against HT29 colorectal adenocarcinoma and HeLa cell lines, and their cytotoxicities are determined at several sensitive positions in the veraguamide scaffold [41]. Six analogues of opunalide, **40**–**42** and veraguamides H–J (**47**–**49**), have been isolated from the marine cyanobacterium cf. *Oscillatoria margaritifera* collected from Coiba Island National Park, Pacific Panama. Compounds **40** and **41** contain bromine, conforming to the structural characteristics of the marine natural products. Compound **40** displays strong cytotoxicity to the H-460 human lung cancer cell lines with LD_50_ value of 141 nM [14]. The total synthesis of the proposed structure for compound **40** has been achieved, but the NMR data of the synthetic compound were significantly different from the natural product **40** [42].

**Figure 9:**
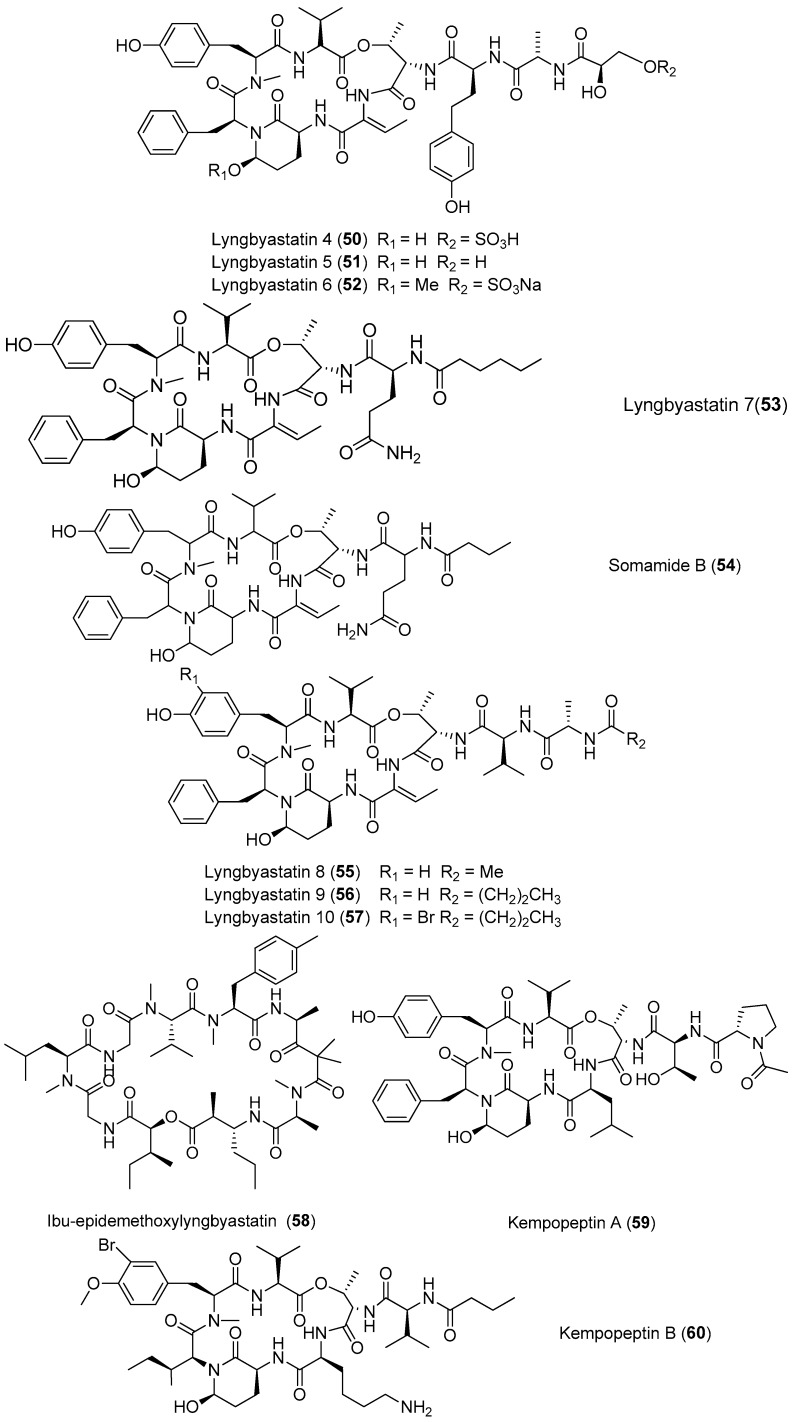
Chemical structures of compounds **50**–**60**.

A large group of cyclic depsipeptides (lyngbyastatins) with various selectivity for elastase, chymotrypsin and trypsin has been purified from marine cyanobacteria, mainly from *Lyngbya* species (Figure 9). Three novel analogues of dolastatin 13, lyngbyastatin 4–6 (**50**–**52**) have been identified from the marine cyanobacterium *Lyngbya confervoides* from the Florida Atlantic coast and South Florida, and the presence of compounds **50**–**52** further supports the conclusion that cyanobacteria are the real origin of many dolastatins. Compound **50** shows potent and selective inhibitory effects on elastase as well as chymotrypsin in vitro over other serine proteases with IC_50_ values of 0.03 and 0.30 μM, respectively [32,43,44]. Another two novel analogues of dolastatin 13, lyngbyastatin 7 (**53**) and somamide B (**54**) have been purified from *Lyngbya* sp. from Florida. Compounds **51**–**54** show potent and selective inhibitory effects on porcine pancreatic elastase over some other serine proteases with IC_50_ values ranging from 3 to 10 nM [44]. The total synthesis of **53** has been completed [45]. Three cyclic depsipeptides with potent elastase inhibitory activity, termed lyngbyastatins 8–10 (**55**–**57**), have been isolated from the marine cyanobacterium *Lyngbya semiplena* collected in Tumon Bay, Guam. Like **50**–**53**, compounds **55**–**57** show strong inhibitory activity against porcine pancreatic elastase with IC_50_ values of 123, 210 and 120 nM, respectively [46]. Ibu-epidemethoxylyngbyastatin 3 (**58**) has been purified from the marine cyanobacterium *Leptolyngbya* sp. from the *SS Thistlegorm* shipwreck in the Red Sea. Compound **58** shows weak cytotoxicity to neuro-2a cells (IC_50_ > 10 μM) [47]. Two analogues of lyngbyastatin, named kempopeptins A and B (**59**, **60**), have been extracted from the marine cyanobacterium *Lyngbya* sp. from Florida. Compound **59** exhibits inhibitory activities against elastase and chymotrypsin (IC_50_ values of 0.32 and 2.6 μM, respectively), while **60** inhibits trypsin (IC_50_ value of 8.4 μM) [48].

**Figure 10:**
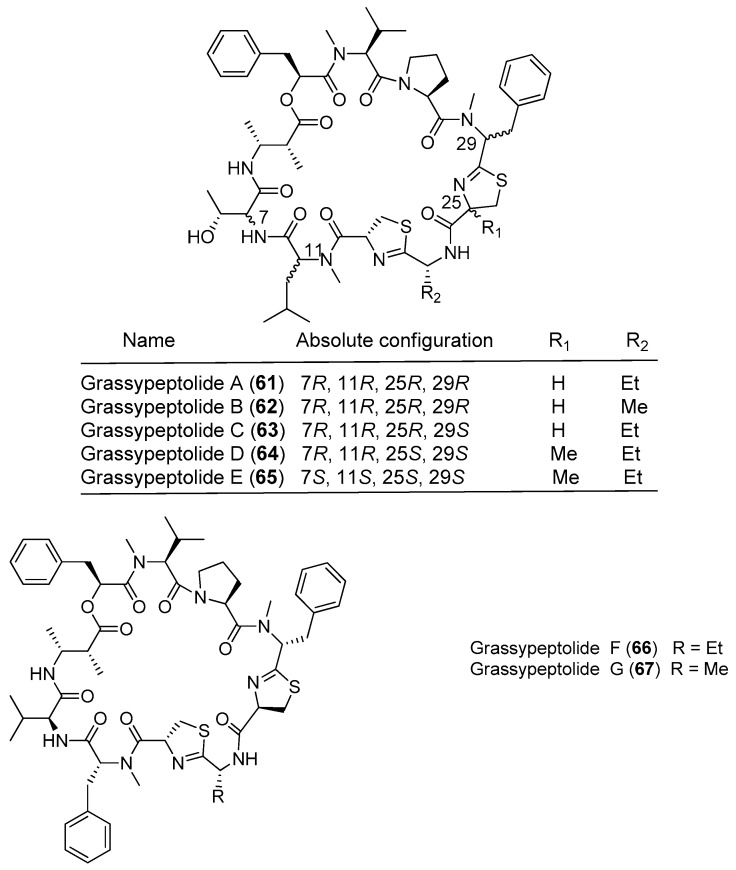
Chemical structures of compounds **61**–**67**.

As a novel family of bis-thiazoline-containing macrocyclic depsipeptides, grassypeptolides containing d-amino acid residues and *β*-amino acid residues have been isolated from marine cyanobacteria (Figure 10). Grassypeptolide A (**61**) has been purified from the marine cyanobacterium *Okeania lorea* (formerly *Lyngbya confervoides*) off Grassy Key in Florida [9], and it inhibits the growth of four cancer cell lines with IC_50_ values ranging from 1.0 to 4.2 μM [49]. Two analogues of compound **61**, grassypeptolides B and C (**62**, **63**), have been extracted from the marine cyanobacterium *Okeania lorea* (formerly *Lyngbya confervoides*) from the Florida Keys [9]. The structure–activity relationship between the analogues shows that when the ethyl substituent of compound **61** is changed to a methyl substituent in **62**, cytotoxic activity is only slightly reduced (3–4-fold), whereas inversion of the Phe unit flanking the bis-thiazoline moiety results in 16–23-fold greater potency. Both compounds **61** and **63** cause cell cycle arrest in G1 phase at lower concentrations, followed by G2/M phase arrest at higher concentrations, and these compounds bind Cu^2+^ and Zn^2+^ [50]. Total synthesis of compound **61** has been accomplished [51]. Grassypeptolides D and E (**64**, **65**) have been derived from the marine cyanobacterium *Leptolyngbya* sp. collected from the *SS Thistlegorm* shipwreck in the Red Sea, and they show significant cytotoxicity against HeLa cell lines (IC_50_ = 335 and 192 nM, respectively) and mouse neuro-2a blastoma cells (IC_50_ = 599 and 407 nM, respectively) [47]. Grassypeptolides F and G (**66**, **67**) have been purified from Palauan cyanobacterium *Lyngbya majuscula*, and they (**66**, **67**) moderately inhibit the transcription factor AP-1 with IC_50_ values of 5.2 and 6.0 μM, respectively [52].

**Figure 11:**
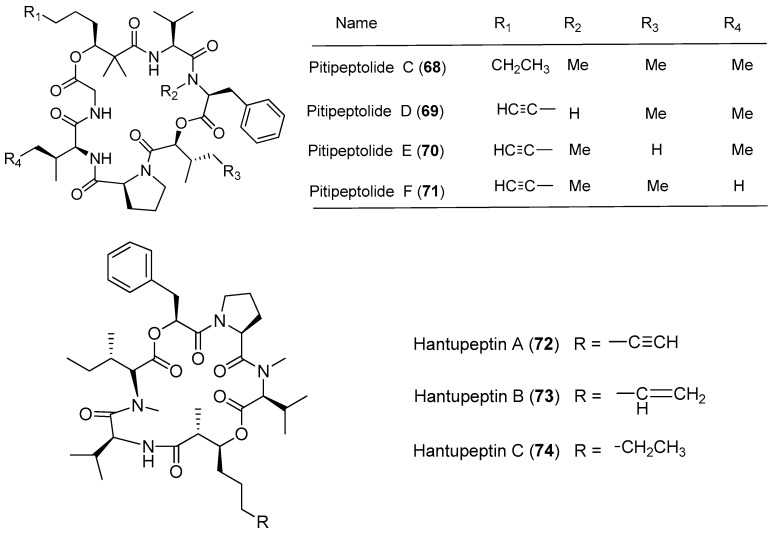
Chemical structures of compounds **68**–**74**.

**Figure 12:**
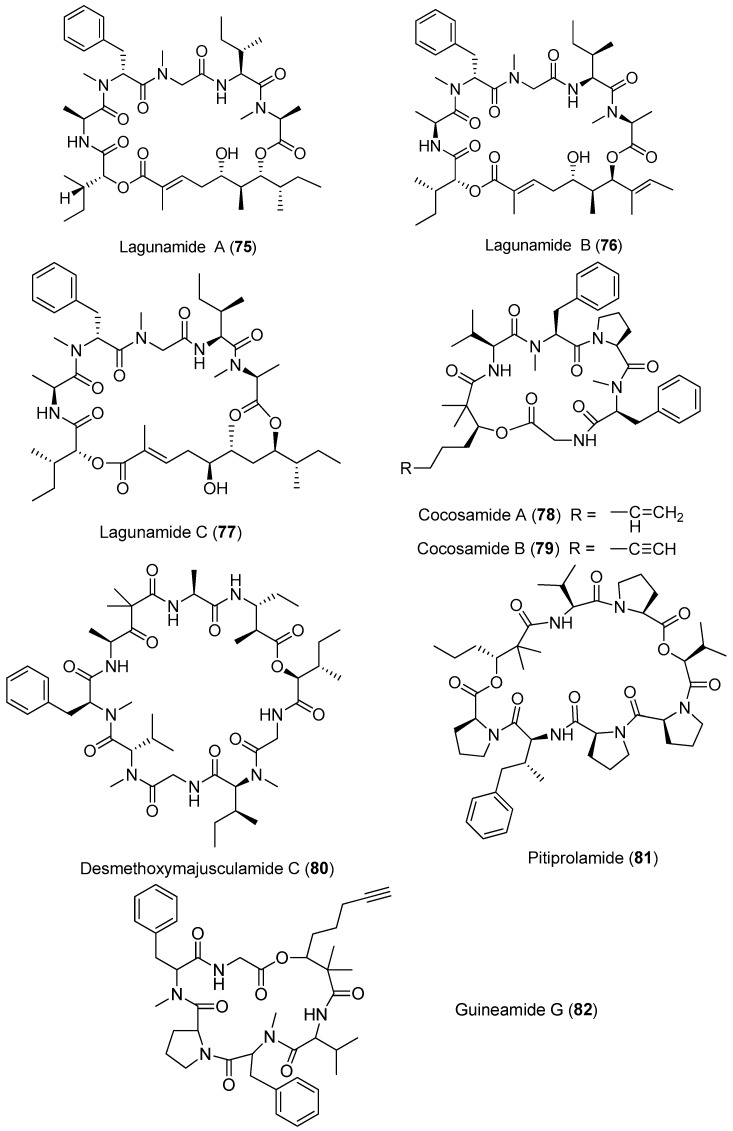
Chemical structures of compounds **75**–**82**.

*Lyngbya majuscula* has been proved to be a chemically prolific species of cyanobacterium since a large number of natural products with structural diversity have been purified from *Lyngbya majuscula* (Figure 11 and Figure 12). Pitipeptolides C–F (**68**–**71**) are antimycobacterial cyclodepsipeptides isolated from the marine cyanobacterium *Lyngbya majuscula* from Piti Bomb Holes, Guam (Figure 11). They (**68**–**71**) show weak cytotoxicity against HT-29 colon adenocarcinoma and MCF7 breast cancer cells [53]. Three cytotoxic cyclic depsipeptides, hantupeptins A–C (**72**–7**4**), have been derived from the marine cyanobacterium *Lyngbya majuscula* from Pulau Hantu Besar, Singapore [54,55]. Compound **72** shows strong cytotoxicity against leukemia cells and breast cancer MCF-7 cell (IC_50_ values of 32 and 4.0 μM, respectively) [54], while compounds **73** and **74** display moderate cytotoxicity against MOLT-4 (leukemia) and MCF-7 cell lines [55].

Three new cytotoxic cyclic depsipeptides, lagunamides A–C (**75**–**77**), have been extracted from *Lyngbya majuscula* collected from Pulau Hantu Besar, Singapore (Figure 12). Compounds **75**–**77** show antimalarial activity against *Plasmodium falciparum* with IC_50_ values of 0.19, 0.91 and 0.29 μM, respectively. Compounds **75** and **76** display significant cytotoxicity against P388 leukemia cells with IC_50_ values of 6.4 and 20.5 nM, respectively. Compound **77** exhibits potent cytotoxicity against several cancer cell lines, such as P388, A549, PC3, HCT8 and SK-OV3 cell lines, with IC_50_ values ranging from 2.1 to 24.4 nM. Furthermore, these compounds **75**–**77** display anti-swarming activities against *Pseudomonas aeruginosa* PA01 [56,57]. Total synthesis of **75** has been achieved, leading to revision of the structure of compound **75** [58].

Two cyclic depsipeptides, cocosamides A and B (**78**, **79**), have been purified from marine cyanobacterium *Lyngbya majuscula* collected from the Cocos Lagoon, Guam, and they display weak cytotoxicity against MCF-7 breast cancer cells and HT-29 colon cancer cells [59]. A potent cytotoxic cyclic depsipeptide, desmethoxymajusculamide C (**80**) extracted from *Lyngbya majuscula* from the Fijian, displays potent and selective cytotoxicity against the HCT-116 human colon carcinoma cell line with an IC_50_ value of 20 nM [60]. A proline-rich analogue of dolastatin 16, pitiprolamide (**81**) isolated from *Lyngbya majuscula* collected from Guam, exhibits weak cytotoxicity against HCT116 colorectal cancer and MCF-7 breast cancer cell lines, and weak antibacterial activity against *Mycobacterium tuberculosis* and *Bacillus cereus* [61]. Guineamide G (**82**) has been extracted from *Lyngbya majuscula*. Compound **82** exhibits brine shrimp toxicity and shows potent cytotoxicity against mouse neuroblastoma cell line with an LC_50_ value of 2.7 μM [62].

**Figure 13:**
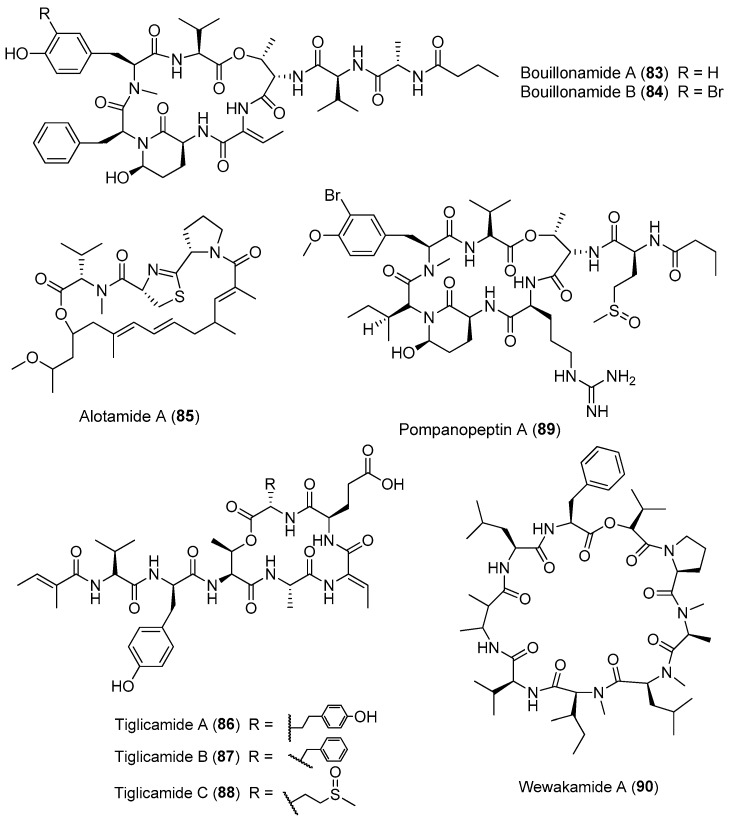
Chemical structures of compounds **83**–**90**.

Except for *Lyngbya majuscula*, other cyanobacteria of the genus *Lyngbya* are important producers of bioactive cyclic depsipeptides (Figure 13). Two proteases inhibitors, bouillomides A and B (**83**, **84**), have been isolated from the marine cyanobacterium *Lyngbya bouillonii* from Guam. Compounds **83** and **84** selectively inhibit serine protease elastase (IC_50_ = 1.9 μM for both) and chymotrypsin (IC_50_ = 0.17 and 9.3 μM, respectively), whereas no trypsin inhibition has been found (IC_50_ > 100 μM) [63]. A structurally intriguing cyclic depsipeptide, alotamide A (**85**) containing rare unsaturated heptaketide residues, has been extracted from *Lyngbya bouillonii* collected from Papua New Guinea. Compound **85** shows an unusual calcium influx in murine cerebrocortical neurons with an EC_50_ value of 4.18 μM [64]. Three protease inhibitors, named tiglicamides A–C (**86**–**88**) containing an unusual tiglic acid moiety, have been purified from the marine cyanobacterium *Lyngbya confervoides* from Florida. Compounds **86**–**88** display moderate inhibitory effects on porcine pancreatic elastase with IC_50_ values ranging from 2.14 to 7.28 μM [65]. Pompanopeptin A (**89**) has been purified from *Lyngbya confervoides* from the coast of Florida. Compound **89** containing an arginine residue in the cyclic core shows selective protease inhibition of trypsin (IC_50_ value of 2.4 μM) over elastase and chymotrypsin [66]. Wewakamide A (**90**) has been extracted from *Lyngbya semiplena* and exhibits brine shrimp toxicity [62].

**Figure 14:**
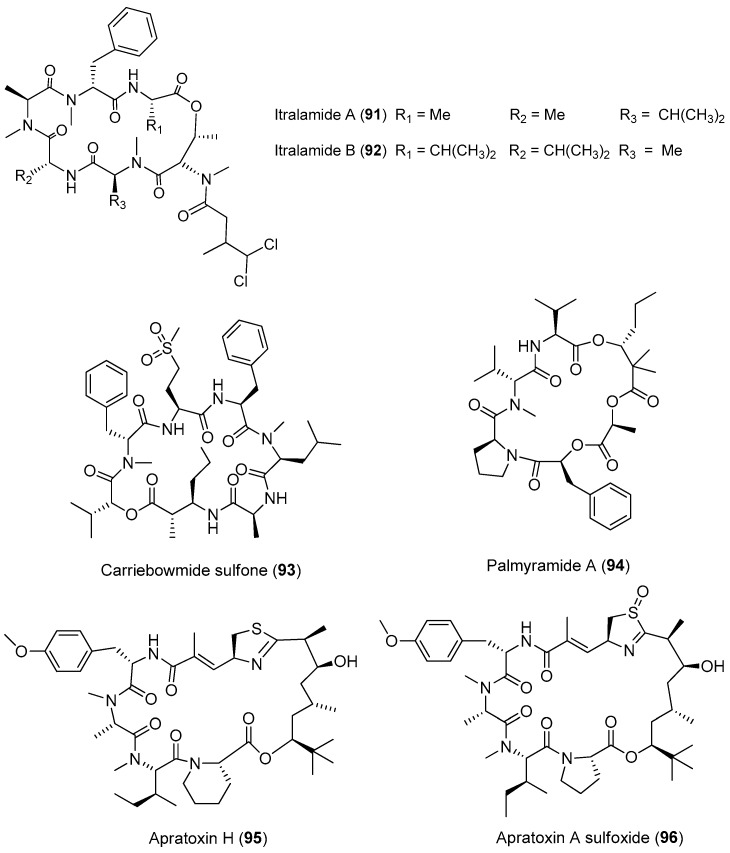
Chemical structures of compounds **91**–**96**.

Three new cytotoxic cyclic depsipeptides, named itralamides A and B (**91**, **92**) and carriebowmide sulfone (**93**), have been extracted from *Moorea producens* (formerly *Lyngbya majuscula*) from the Eastern Caribbean (Figure 14). Only compound **92** exhibits significant cytotoxicity with an IC_50_ value of 6 μM [24,67]. The total synthesis of the proposed structure for **92** has been achieved, but the true structure of the natural compound **92** is still unknown [68].

A cyclic depsipeptide, palmyramide A (**94**), has been identified through bioassay-guided isolation of the marine cyanobacterium *Moorea producens* (formerly *Lyngbya majuscula*) from Palmyra Atoll [24]. Compound **94** displays sodium channel-blocking activity in neuro-2a cells (IC_50_ value of 17.2 μM) and shows moderate cytotoxicity in H-460 human lung carcinoma cells (IC_50_ value of 39.7 μM) [69]. Cultivation of the *Moorea producens* (formerly *Lyngbya majuscula*) affords two new apratoxin analogues, termed apratoxin H (**95**) and apratoxin A sulfoxide (**96**). Compounds **95** and **96** exhibit strong cytotoxicity against human NCI-H460 lung cancer cells with IC_50_ values of 3.4 and 89.9 nM, respectively. The biosynthetic pathway of apratoxins supports the opinion that structural diversity of apratoxin can be the result of regional diversity [70].

**Figure 15:**
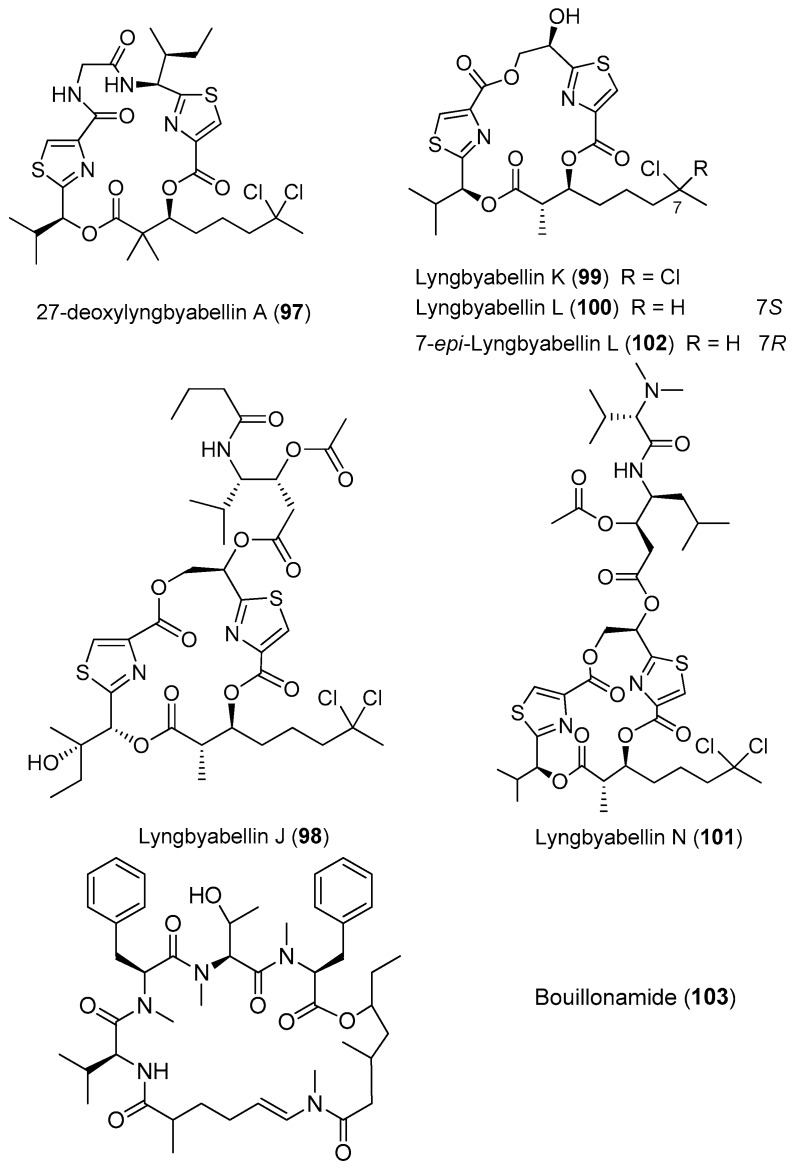
Chemical structures of compounds **97**–**103**.

Two analogues of lyngbyabellin A, 27-deoxylyngbyabellin A (**97**) and lyngbyabellin J (**98**), have been isolated from *Moorea bouillonii* (formerly *Lyngbya bouillonii*) collected from shallow patch reefs in Apra Harbor, Guam [8]. Compounds **97** and **98** show weak cytotoxicity against HT29 colon cancer cells and HeLa cervical cancer cells [26]. Four novel cyclic petides, lyngbyabellins K and L (**99**, **100**), lyngbyabellin N (**101**) and 7-*epi*-lyngbyabellin L (**102**), have been extracted from two collections of marine cyanobacteria *Moorea bouillonii* from Palmyra Atoll in the Central Pacific Ocean (Figure 15). Compound **101** containing an unusual *N*,*N*-dimethylvaline residue and a leucine statine residue shows potent cytotoxicity against HCT116 colon cancer cell line with an IC_50_ value of 40.9 nM [37]. A novel cytotoxic cyclic depsipeptide, bouillonamide (**103**), has been derived from the tropical marine cyanobacterium *Moorea bouillonii* collected from New Britain, Papua New Guinea. Compound **103** shows mild toxicity against the neuron 2a mouse neuroblastoma cells with IC_50_ value of 6.0 μM [71].

**Figure 16:**
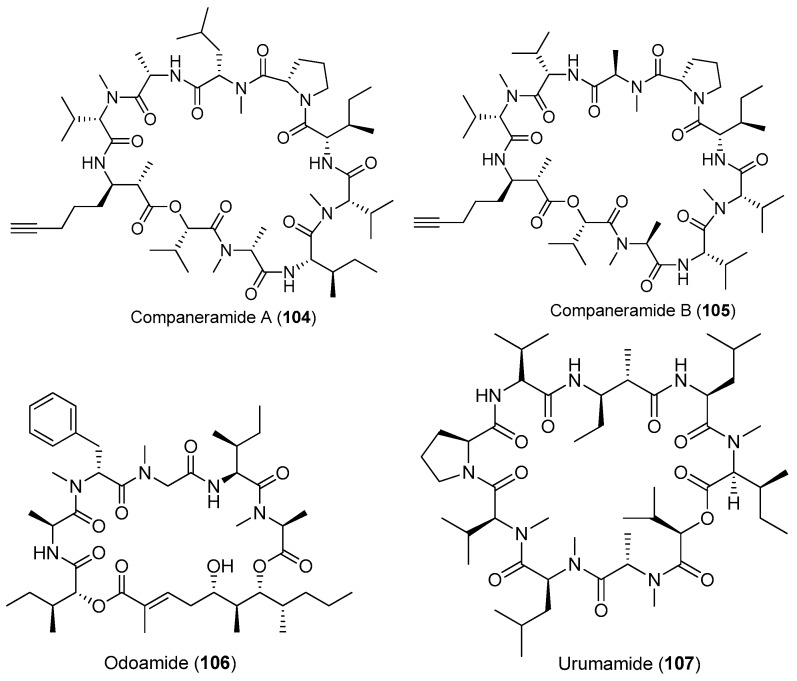

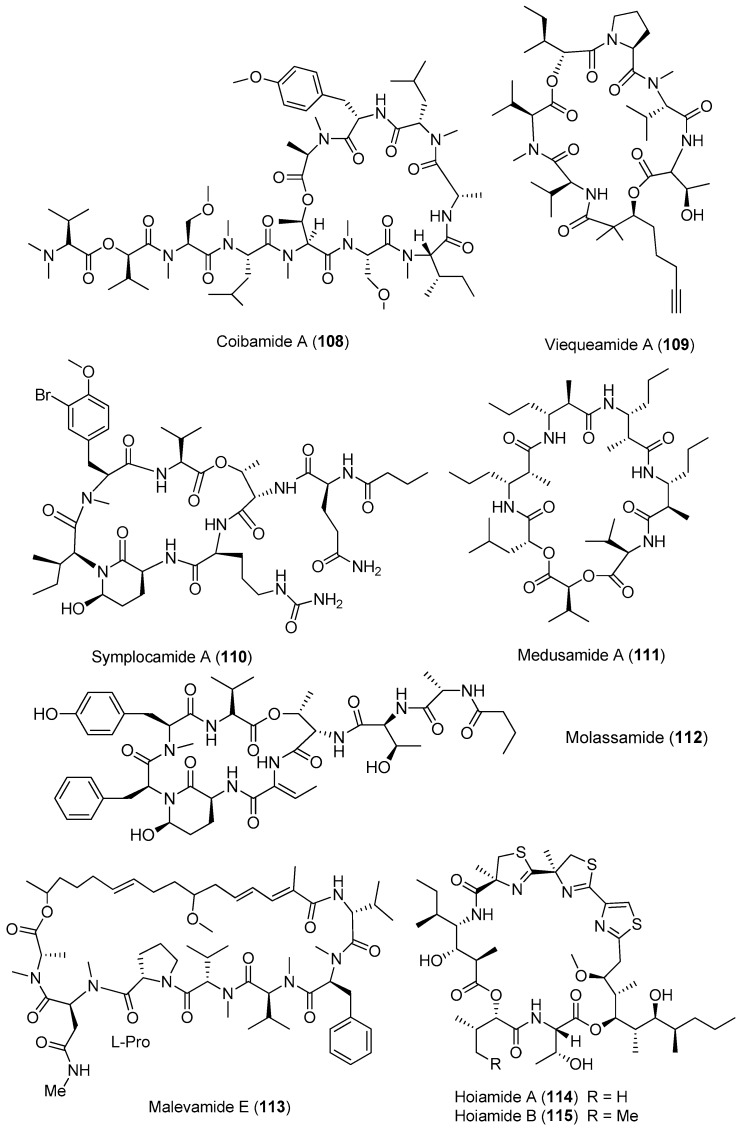
Chemical structures of compounds **104**–**115**.

Except for cyanobacteria of the genera *Lyngbya* and *Moorea*, other species of marine cyanobacteria also have provided some bioactive cyclic depsipeptides (Figure 16). Two antimalarial cyclodepsipeptides, companeramides A and B (**104**, **105**), have been isolated from the marine cyanobacterial assemblage collected from Coiba Island, Panama. Compounds **104** and **105** display high antiplasmodial activity [72]. Two new cyclic cyclodepsipeptides, odoamide (**106**) and urumamide (**107**), have been purified from *Okeania* sp. collected from Okinawa Prefecture and Ikei Island, Okinawa, respectively [73,74]. Compound **106** shows strong cytotoxicity against HeLa S3 human cervical cancer cells with an IC_50_ of 26.3 nM [73]. Compound **107** inhibits the growth of HeLa and HL60 cells with IC_50_ values of 18 and 13 μM, respectively. Compound **107** inhibits chymotrypsin with an IC_50_ value of 33 μM [74]. An unprecedented cytotoxic depsipeptide, coibamide A (**108**), has been identified from *Caldora penicillata* (formerly *Leptolyngbya* sp.) from Coiba Island National Park, Panama (Figure 16). Compound **108** contains eight *N*-methylated amino acid residues and shows significant and selective cytotoxicity against NCI-H460 lung cancer cells and mouse neuro-2a cells [10,75]. The structure of compound **108** has been revised by successful total synthesis [76]. A new marine cyclicdepsipeptide with potent cytotoxicity, viequeamide A (**109**), has been derived from the marine cyanobacterium *Rivularia* sp. from the Vieques Island, Puerto Rico. Compound **109** displays potent cytotoxicity against H460 human lung cancer cells with an IC_50_ value of 60 nM [77]. Total synthesis of **109** has been completed [78].

A potent cytotoxin, symplocamide A (**110**), has been identified from *Symploca* sp. collected from Papua New Guinea. Compound **110** shows potent cytotoxicity against H-460 lung cancer cells and neuro-2a neuroblastoma cells with IC_50_ values of 40 and 29 nM, respectively. Compound **110** selectively inhibits chymotrypsin with a greater potency (200-fold) than trypsin [79]. The first cyclic depsipeptide reported to contain multiple Amha residues and four contiguous *β*-amino acid residues, named medusamide A (**111**), has been isolated from a collection of marine cyanobacteria from Coiba Island on the Pacific coast of Panama [80]. The first peptide from the genus *Dichothrix*, termed molassamide (**112**), has been purified from the marine cyanobacterium *Dichothrix utahensis* collected from the Molasses Reef, Key Largo, Florida and from Brewer’s Bay, St. Thomas, U.S. Virgin Islands. Compound **112** exhibits serine protease inhibition against elastase and chymotrypsin with IC_50_ values of 0.032 and 0.234 μM, respectively. No inhibitory activity against trypsin has been found at the highest concentration tested (10 μM) [81]. Malevamide E (**113**) isolated from the marine cyanobacterium *Symploca laeteviridis* shows store-operated Ca^2+^ entry in thapsigargin-treated human embryonic kidney (HEK) cells with a dose-dependent inhibition manner (2–45 μM) [82].

Hoiamide A (**114**), isolated from Papua New Guinea marine cyanobacteria *Lyngbya majuscula* and *Phormidium gracile*, is an unusual cyclic depsipeptide that consists of an acetate extended and S-adenosyl methionine modified isoleucine moiety, a triheterocyclic fragment bearing two α-methylated thiazolines and one thiazole, and a highly oxygenated and methylated C15-polyketide substructure (Figure 16). Compound **114** is potent inhibitor of voltage-gated sodium channels (IC_50_ = 92.8 nM), and it can activate sodium influx (EC_50_ = 2.31 μM) in mouse neocortical neurons [83]. An analogue of compound **114**, hoiamide B (**115**), has been derived from two different collections of marine cyanobacteria from Papua New Guinea. Compound **115** stimulates sodium influx and suppresses spontaneous Ca^2+^ oscillations in neocortical neurons with EC_50_ values of 3.9 μM and 79.8 nM, respectively [34].

### 3.2. Other Cyclic Peptides

**Figure 17:**
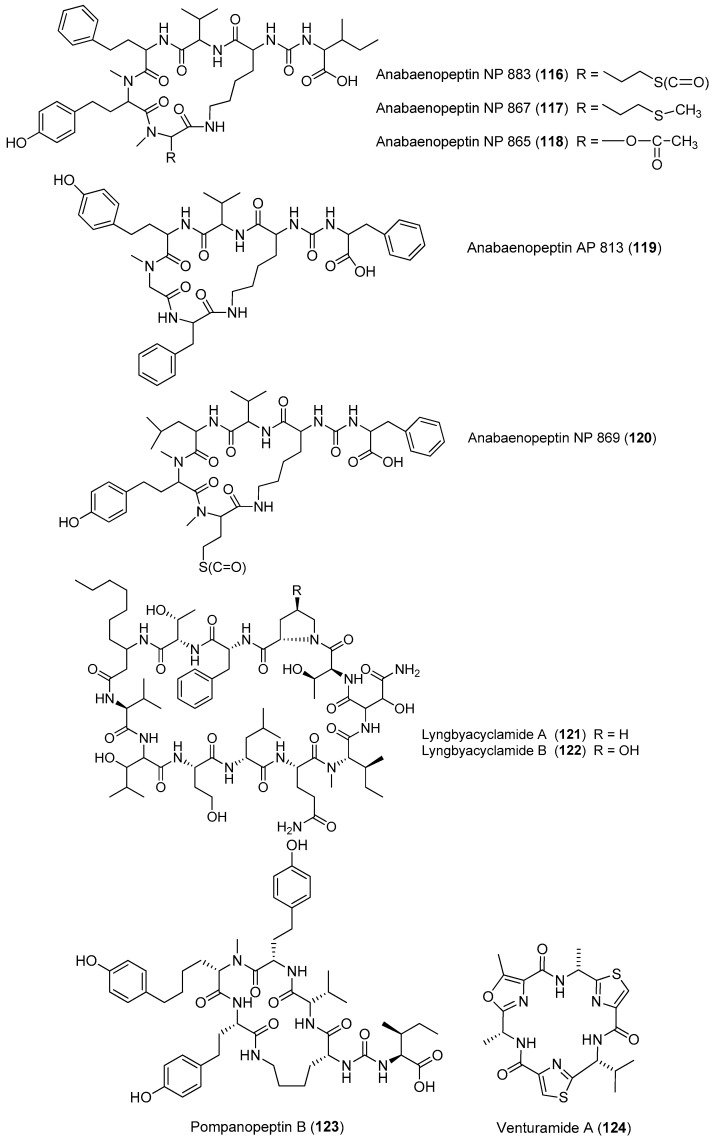

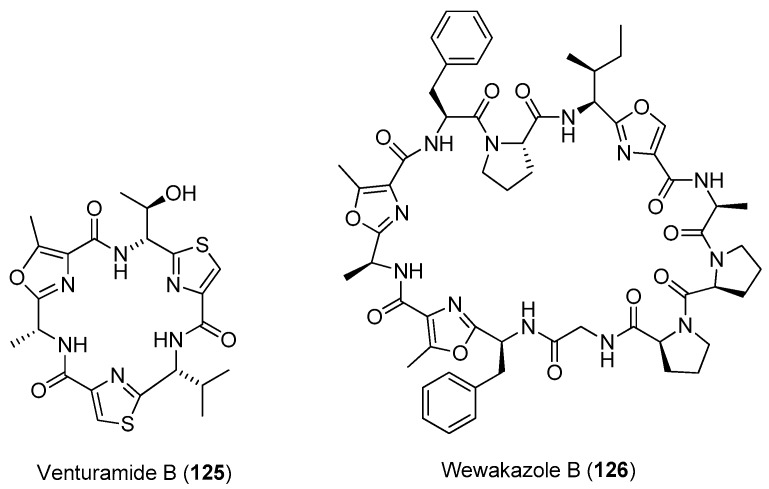
Chemical structures of compounds **116**–**126**.

Five anabaenopeptin-related compounds (called by their molecular weight), termed anabaenopeptin NP883 (**116**), anabaenopeptin NP867 (**117**), anabaenopeptin NP865 (**118**), anabaenopeptin AP813 (**119**) and anabaenopeptin NP869 (**120**), have been isolated from a bloom sample of marine cyanobacteria of Baltic Sea (Figure 17). Compounds **116**–**120** inhibit carboxypeptidase A and protein phosphatase 1 with varying potency [84]. Two new cyclic peptides, lyngbyacyclamides A and B (**121**, **122**), have been purified from the marine cyanobacterium *Lyngbya* sp. collected from Okinawa Prefecture, Japan. Compounds **121** and **122** show cytotoxicity against the growth of B16 melanoma cells with an IC_50_ of 0.7 μM [85]. Total synthesis of **121** has been completed [86]. Pompanopeptin B (**123**) has been purified from *Lyngbya confervoides* from the coast of Florida [66]. Two antimalarial cyclic hexapeptides, venturamides A and B (**124**, **125**), have been identified from the Panamanian marine cyanobacterium *Oscillatoria* sp. through antimalarial bioassay-guided isolation [87]. A new cyanobacterial toxin, wewakazole B (**126**), has been isolated from *Moorea producens* collected in the Red Sea by mass spectrometry-guided isolation. Compound **126** shows cytotoxicity against human MCF7 breast cancer cells and human H460 lung cancer cells with IC_50_ values of 0.58 and 1.0 μM, respectively [88]. Total synthesis of **126** has been achieved [89].

## 4. Conclusions

Marine cyanobacteria are the significant sources of structurally diverse marine natural products with broad biological activities. Significant progress has been made in discovery of bioactive secondary metabolites from marine cyanobacteria over the past decade. The overwhelming majority of cyanobacterial secondary metabolites are peptides, especially cyclic depsipeptides (76 compounds), accounting for more than half of the total cyanobacterial peptides (126 compounds).

However, there are lots of problems in drug development from marine cyanobacteria, including evaluation of the taxonomy of cyanobacteria, new techniques developed to culture marine cyanobacteria in mass, total synthesis and multi-target screening assay. Firstly, the cyanobacterial genus *Lyngbya*, especially *Lyngbya majuscula*, has been proved to be important producers of marine peptides. Three new cyanobacterial genera *Moorea*, *Okeania* and *Caldora*, have been proposed in the past few years. The three new genera were previously identified as the chemically rich genera *Lyngbya* and *Symploca*, respectively [8,9,10]. Moreover, several important peptides with promising pharmaceutical potential, such as symplostatin 1 and dolastatin 10, were actually isolated from these new genera of cyanobacteria. At present, the chemically rich genus *Lyngbya* has been shown to be polyphyletic, and biodiversity in tropical marine cyanobacteria remains currently unclear [10]. It is necessary to evaluate the taxonomy of NP-rich marine cyanobacteria using a combined molecular, morphological and chemical approach in further research.

Secondly, cyanobacteria have great potential as sustainable sources for production of bioactive peptides because of their rapid growth, genetic tractability and cultivable property [3]. Although cyanobacteria possess the cultivable properties similar to those of microorganisms, cyanobacteria have attracted far less attention than microorganisms. More efforts should be invested in developing new techniques to culture marine cyanobacteria in mass. Thirdly, total synthesis of some bioactive cyanobacterial peptides has been successfully achieved, which might be beneficial for the structure revision of natural peptides, further evaluation and pharmacological applications. In addition, at present, the majority of cyanobacterial peptides exhibit a broad range of bioactivities, including cytotoxic, antibacterial, antimalarial, enzyme inhibition, parasitic resistance and channel-blocking activities. The overwhelming majority of cyanobacterial peptides display in vitro antitumor activity. Multi-target screening assays should be developed to accelerate the discovery of promising drug leader compounds.

At present, two scholars, including Luesch H. and Gerwick W., have greatly contributed to the discovery of new peptides from marine cyanobacteria. Programs for drug discovery from marine cyanobacteria, such as the Panama ICBG program, have led to the discovery of bioactive cyanobacterial peptides. Marine cyanobacteria have great potential as sustainable marine sources for production of bioactive peptides (such as dolastatins) because of their genetic tractability, cultivable property, rapid growth and peptide biosynthetic pathway. This review summarized new peptides derived from marine cyanobacteria over the past decade, providing useful information in the further discovery of novel cyanobacterial peptides.

## Figures and Tables

**Table 1:** Bioactivities of linear depsipeptides from marine cyanobacteria.

Metabolites	Sources	Activities	References
Grassystatins A–B (**1**–**2**)	*Okeania lorea* (formerly *Lyngbya* cf. *confervoides*)	Cathepsin inhibition	[12,13]
Veraguamides K–L (**3**–**4**)	cf. *Oscillatoria margaritifera* Coiba, Panama	nd ^a^	[14]
Maedamide (**5**)	*Lyngbya* sp.	Potent antitumor cytotoxicity Protease inhibition	[15,16]
Viridamides A–B (**6**–**7**)	*Okeania comitata* (formerly *Oscillator nigroviridis*) Panama	Antitrypanosomal activity Antileishmanial activity	[17]
Gallinamide A (**8**)	*Schizothrix* iedras Gallinas	Antimalarial activity	[18,19]

^a^ Not determined.

**Table 2:** Bioactivities of linear peptides from marine cyanobacteria.

Metabolites	Sources	Bioactivities	References
Almiramides A–C (**9**–**11**)	*Lyngbya majuscula* Panama	General antileishmanial activity Antitumor cytotoxicity	[20,21]
Almiramide D (**12**)	*Oscillatoria nigroviridis* Island of Providence (Colombia, S.W. Caribbean Sea)	Antitumor cytotoxicity	[21]
Almiramide E–H (**13**–**16**)	*Oscillatoria nigroviridis* Island of Providence (Colombia, S.W. Caribbean Sea)	nd ^a^	[21]
Dragonamides A–B (**17**–**18**) Carmabin A (**19**) Dragomabin (**20**)	*Moorea producens* (formerly *Lyngbya polychroa*) Panama	Antimalarial activity	[22]
Dragonamides C–D (**21**–**22**)	*Moorea producens* (formerly *Lyngbya polychroa*) Florida, Fort Lauderdale, Hollywood	Weak antitumor cytotoxicity	[23,24]
Dragonamide E (**23**)	*Lyngbya majuscula*	Antileishmanial activity	[25]
Lyngbyapeptin D (**24**)	*Moorea bouillonii* (formerly *Lyngbya bouillonii*) Apra Harbor, Guam	nd ^a^	[26]
Jahanyne (**25**)	*Lyngbya* sp.	Potent antitumor cytotoxicity	[27]
Bisebromoamide (**26**)	*Lyngbya* sp.	Protein kinase inhibition	[28,29,30,31]
Norbisebromoamide (**27**)	*Lyngbya* sp.	nd ^a^	[29]
Tasiamides C–D (**28**–**29**)	*Symploca* sp. Papua New Guinea	Weak antitumor cytotoxicity	[32]
Tasiamide E (**30**)	*Symploca* sp. Papua New Guinea	nd ^a^	[32]
Carmaphycins A–B (**31**–**32**)	*Symploca* sp. Curacao	Protease inhibition Potent antitumor cytotoxicity	[33]
Hoiamides C–D (**33**–**34**)	Cyanobacterium Papua New Guinea	nd ^a^	[34,35,36]
Lyngbyabellin M (**35**)	cyanobacterium from Palmyra Atoll Central Pacific Ocean	nd ^a^	[37]
Kurahyne (**36**)	cyanobacterial mixture	Antitumor cytotoxicity	[38]
Kurahyne B (**37**)	*Okeania* sp.	Mild antitumor cytotoxicity	[39]
Caldoramide (**38**)	*Caldora penicillata* Florida	Antitumor cytotoxicity	[40]
Grassystatin C (**39**)	*Okeania lorea* (formerly *Lyngbya* cf. *confervoides*)	Cathepsin inhibition	[12]

^a^ Not determined.

**Table 3:** Bioactivities of cyclic depsipeptides (**40**–**67**) from marine cyanobacteria.

Structure Class	Metabolites	Sources	Activities	References
Veraguamides	Veraguamides A–G (**40**–**46**)	cf*. Oscillatoria margaritifera*, Panama *Symploca* cf. *hydnoides* Cetti Bay, Guam	Weak antitumor cytotoxicity	[14,41,42]
	Veraguamides H–J (**47**–**49**)	cf*. Oscillatoria margaritifera* Panama	nd ^a^	[14]
Lyngbyastatins	Lyngbyastatins 4–6 (**50**–**52**)	*Lyngbya confervoides* off the coast of Florida	Potent protease inhibition	[43,44]
	Lyngbyastatin 7 (**53**) Somamide B (**54**)	*Lyngbya* sp. from Florida	Potent protease inhibition	[44,45]
	Lyngbyastatins 8–10 (**55**–**57**)	*Lyngbya semiplena* Tumon Bay, Guam	Potent protease inhibition	[46]
	Ibu-epidemethoxylyngbyastatin 3 (**58**)	*Leptolyngbya* sp. *SS Thistlegorm* shipwreck, Red Sea	Weak cytotoxicity to neuro-2a cells	[47]
	Kempopeptins A and B (**59**, **60**)	*Lyngbya* sp. Florida	Potent protease inhibition	[48]
	Grassypeptolide A (**61**)	*Okeania lorea* (formerly *Lyngbya confervoides*) off Grassy Key in Florida	Antitumor cytotoxicity	[49]
	Grassypeptolides A–C (**61**–**63**)	*Okeania lorea* (formerly *Lyngbya confervoides*)	Cause G1 and G2/M phase cell cycle arrest	[50,51]
Grassypeptolides	Grassypeptolides D and E (**64**, **65**)	*Leptolyngbya* sp. *SS Thistlegorm* shipwreck, Red Sea	Potent antitumor cytotoxicity	[47]
	Grassypeptolides F and G (**66**, **67**)	*Lyngbya majuscula* Panama	Moderate inhibitory activity against the transcription factor AP-1	[52]

**Table 4:** Bioactivities of cyclic depsipeptides (**68**–**115**) from marine cyanobacteria.

Sources	Metabolites	Sources/Location	Activities	References
*Lyngbya majuscula*	Pitipeptolides C–E (**68**–**70**)	Guam, Piti Bomb Holes	nd ^a^	[53]
	Pitipeptolide F (**71**)	Guam, Piti Bomb Holes	Antibacterial activity	[53]
	Hantupeptins A–C (**72**–**74**)	Pulau Hantu Besar Singapore	Moderate antitumor cytotoxicity	[54,55]
	Lagunamides A–C (**75**–**77**)	Pulau Hantu Besar Singapore	Antimalarial activity Potent antitumor cytotoxicity	[56,57,58]
	Cocosamides A and B (**78**, **79**)	Cocos Lagoon, Guam	Slight antitumor cytotoxicity	[59]
	Desmethoxymajusculamide C (**80**)	Fijian	Potent antitumor cytotoxicity	[60]
	Pitiprolamide (**81**)	Piti Bomb Holes, Guam	Weak antitumor cytotoxicity Weak antibacterial activity	[61]
	Guineamide G (**82**)	Papua New Guinea	Brine shrimp toxicity Cytotoxicity against neuroblastoma cell	[62]
Genus *Lyngbya*	Bouillomides A and B (**83**, **84**)	*Lyngbya bouillonii*, Guam	Protease inhibition	[63]
	Alotamide A (**85**)	*Lyngbya bouillonii* Papua New Guinea	Influx of Ca^2+^ in murine cerebrocortical neurons	[64]
	Tiglicamides A–C (**86**–**88**)	*Lyngbya confervoides* Florida	Protease inhibition	[65]
	Pompanopeptin A (**89**)	*Lyngbya confervoides* Florida	Protease inhibition	[66]
	wewakamide A (**90**)	*Lyngbya semiplena* Papua New Guinea	Potent brine shrimp toxicity	[62]
*Moorea producens*	Itralamides A and B (**91**, **92**)	eastern Caribbean	Antitumor cytotoxicity	[67,68]
	Carriebowmide sulfone (**93**)	eastern Caribbean	nd ^a^	[67]
	Palmyramide A (**94**)	Palmyra Atoll	Blocks sodium channel in neuro-2a cells Antitumor cytotoxicity	[69]
	Apratoxin H (**95**) Apratoxin A sulfoxide (**96**)	Gulf of Aqaba, Nabq Mangrovs	Potent antitumor cytotoxicity	[70]
*Moorea bouillonii*	27-deoxylyngbyabellin A (**97**) Lyngbyabellin J (**98**)	Apra Bay, Guam	Moderate antitumor cytotoxicity	[26]
	Lyngbyabellins K–L, N (**99**–**101**) 7-*epi*-Lyngbyabellin L (**102**)	Palmyra Atoll Central Pacific Ocean	Antitumor cytotoxicity	[37]
	Bouillonamide (**103**)	New Britain, Papua New Guinea	Mild toxicity to neuron 2a cells	[71]
Other marine cyanobacteria	Companeramides A andB (**104**, **105**)	cyanobacterium from Panama	Moderate antimalaria parasites	[72]
	Odoamide (**106**)	*Okeania* sp.	Potent antitumor cytotoxicity	[73]
	Urumamide (**107**)	*Okeania* sp.	Mild antitumor cytotoxicity	[74]
	Coibamide A (**108**)	*Caldora penicillata* (formerly *Leptolyngbya* sp.) Panama	Antitumor cytotoxicity	[75,76]
	Viequeamide A (**109**)	*Rivularia* sp. viequeamides uerto Rico, Vieque	Potent antitumor cytotoxicity	[77,78]
	Symplocamide A (**110**)	*Symploca* sp. Papua New Guinea	Potent antitumor cytotoxicity	[79]
	Medusamide A (**111**)	cyanobacterium from Panama	nd ^a^	[80]
	Molassamide (**112**)	*Dichothrix utahensis* Molasses Reef, Key Largo, Florida	Protease inhibition	[81]
	Malevamide E (**113**)	*Symploca laeteviridis*	Inhibits Ca^2+^ release activated Ca^2+^ (CRAC) channels	[82]
	hoiamide A (**114**)	*Lyngbya majuscula* and *Phormidium gracile* Papua New Guinea	nd ^a^	[83]
	Hoiamide B (**115**)	two different cyanobacterium from Papua New Guinea	Activates sodium chanal in mouse neocortical neurons Suppresses spontaneous Ca^2+^ oscillations in neocortical neurons	[34]

^a^ Not determined.

**Table 5:** Bioactivities of cyclic peptides from marine cyanobacteria.

Metabolites	Sources	Activities	References
Anabaenopeptins NP 883, NP 867, NP 865, AP813, NP 869 (**116**–**120**)	bloom sample of marine cyanobacteria Baltic Sea	nd ^a^	[84]
Lyngbyacyclamides A–B (**121**–**122**)	*Lyngbya* sp. Okinawa, Japan	Moderate antitumor cytotoxicity	[85,86]
Pompanopeptin B (**123**)	*Lyngbya confervoides* Florida	Protease inhibition	[66]
Venturamides A and B (**124**, **125**)	*Oscillatoria* sp.	Antimalaria parasites	[87]
Wewakazole B (**126**)	*Moorea producens* Red Sea	Moderate antitumor cytotoxicity	[88]

^a^ Not determined.
